# The Use of Assisted Reproductive Technology by European Childhood Cancer Survivors

**DOI:** 10.3390/curroncol29080453

**Published:** 2022-08-15

**Authors:** Anja Borgmann-Staudt, Simon Michael, Greta Sommerhaeuser, Marta-Julia Fernández-González, Lucía Alacán Friedrich, Stephanie Klco-Brosius, Tomas Kepak, Jarmila Kruseova, Gisela Michel, Anna Panasiuk, Sandrin Schmidt, Laura Lotz, Magdalena Balcerek

**Affiliations:** 1 Charité-Universitätsmedizin Berlin Germany, Corporate Member of Freie Universität Berlin, Humboldt-Universität zu Berlin, Berlin Institute of Health, Department of Paediatric Oncology, Haematology and Stem Cell Transplantation, 13353 Berlin, Germany; 2 Department of Paediatric Oncology, Masaryk University, University Hospital Brno, 61137 Brno, Czech Republic; 3 Department of Pediatric Hematology and Oncology, 2nd Faculty of Medicine, Charles University and University Hospital Motol, 15006 Prague, Czech Republic; 4 Department of Health Sciences and Medicine, University of Lucerne, 6002 Luzern, Switzerland; 5 Department of Oncology, Hematolgy and Transplantology, Medical University Wroclaw, 50-367 Wrocław, Poland; 6 Medical University Graz, 8036 Graz, Austria; 7 Department of Obstetrics and Gynaecology, Erlangen University Hospital, Friedrich-Alexander University of Erlangen–Nuremberg, 91054 Erlangen, Germany; 8 Berlin Institute of Health (BIH), 10178 Berlin, Germany

**Keywords:** childhood and adolescence, cancer, survivor, ART, offspring, health outcome

## Abstract

CCS often wish to have biological children yet harbour concerns about fertility impairment, pregnancy risks and the general health risks of prospective offspring. To clarify these concerns, health outcomes in survivor offspring born following ART (*n* = 74, 4.5%) or after spontaneous conception (*n* = 1585) were assessed in our European offspring study by descriptive and bivariate analysis. Outcomes were compared to a sibling offspring cohort (*n* = 387) in a 4:1 matched-pair analysis (*n* = 1681). (i) Survivors were more likely to employ ART than their siblings (4.5% vs. 3.7%, *p* = 0.501). Successful pregnancies were achieved after a median of one cycle with, most commonly, *intracytoplasmic sperm injection* (ICSI) using non-cryopreserved oocytes/sperm. (ii) Multiple-sibling births (*p* < 0.001, 29.7% vs. 2.5%), low birth weight (*p* < 0.001; OR = 3.035, 95%-CI = 1.615–5.706), and preterm birth (*p* < 0.001; OR = 2.499, 95%-CI = 1.401–4.459) occurred significantly more often in survivor offspring following ART utilisation than in spontaneously conceived children. ART did not increase the prevalence of childhood cancer, congenital malformations or heart defects. (iii) These outcomes had similar prevalences in the sibling population. In our explorative study, we could not detect an influence on health outcomes when known confounders, such as multiple births, were taken into account.

## 1. Introduction

In recent decades, improved treatment has increased childhood cancer survival across Europe. This has led to a growing number of adults requiring specific life-event counselling, such as family planning. Many individuals of childbearing age with a history of cancer wish to have biological children. Still, they are faced with concerns regarding fertility impairment, potential pregnancy risks and the overall health outcome of their offspring [1,2]. Exposure to high-dose chemotherapy or gonadal irradiation can impair fertility or cause permanent infertility, depending on the type of agent, cumulative dosage, patient age and gender [3]. As a consequence, reduced overall pregnancy and live birth rates in former patients compared to the general population have been reported [4,5]. While some childhood cancer survivors conceive naturally, others require the use of assisted reproductive technology (ART), such as in vitro fertilisation (IVF) and/or intracytoplasmatic sperm injection (ICSI), which represent the most commonly used methods. Survivors who achieve pregnancy may experience a range of associated risks to both mother and foetus [6,7], including an increased risk of spontaneous abortion, stillbirth and preterm birth [8,9,10]. These findings were reported mainly in survivors treated with high-dose abdominal irradiation [11,12]. It is encouraging to note that no increased risk of congenital abnormalities was observed in these offspring [13,14]. To date, the adverse impact of cancer treatment on pregnancy outcomes has only been investigated in selected patient groups based on diagnosis or age at diagnosis; however, effects on the long-term health of survivor offspring remain unclear.

In 2019, a global estimation of more than 7 million ART-conceived children was documented; this number is steadily rising and comprises 2–6% of European births [15]. However, concerns exist regarding the health and development of ART-conceived children [16]. Multiple pregnancies resulting from multiple embryo transfers are associated with low birth weight and preterm birth, which can have long-term health implications [17,18]. However, ART has also been suspected of adverse obstetric and perinatal consequences of single births [19,20], such as increased risk of birth defects [21], cancer [22] and early growth [23]. Further evidence suggests that ART treatment also increases the risk of chronic age-related diseases such as obesity, type 2 diabetes and cardiovascular disease [24,25,26,27]. However, the influence of parental characteristics, including the aetiology of subfertility and infertility, or aspects of specific infertility treatments are unknown. The underlying mechanisms, including epigenetic changes that may occur during preimplantation and the development of uterine reprogramming, are currently under debate [28,29]. Publications examining ART and health issues of childhood cancer survivor offspring are rare [30]. Our study compared: (i) the prevalence of ART utilisation in survivors and their siblings, (ii) perinatal outcomes, malformations, heart defects and cancer in ART-conceived survivor offspring with those conceived spontaneously and (iii) the outcomes of survivor offspring vs. sibling offspring.

## 2. Materials and Methods

### 2.1. Study Design and Participants

The European Offspring Study, an explorative, retrospective cohort study, surveyed both adult childhood cancer survivors and their adult siblings on the health of their biological offspring between 2013 and 2016 in five European countries (Germany, Austria, Czech Republic, Poland and Switzerland). Participants who gave their informed consent were surveyed using a 46-item questionnaire [31], designed to address five health-related sub-areas (diseases, health-related behavior, health-related quality of life, healthcare utilisation and living conditions) as well as socio-demographic information. The study was approved by the local ethics committees of participating centres (lead vote *Charité-Universitätsmedizin Berlin,* EA2/237/05, EA2/103/11). The detailed concept and study methods, including recruitment strategies and participant characteristics, have been described previously [32].

### 2.2. Variables

Our analyses included parental reports on offspring gender, year of birth, gestational age (categorized as per *World Health Organization* (WHO)), birth weight (categorized as per WHO), mode of conception, multiple births, diagnoses of congenital malformations and heart defects (both categorized as per *International Classification of Disease* (*ICD-10*)) [33], diagnoses of cancers (categorized as per *The International Classification of Childhood Cancer* (ICCC-3)). Parental characteristics were assessed, including educational attainment (classified as per ISCED [34], country of origin and the family’s migrant background (fulfilled if the offspring, the parents or grandparents were born in another country than the country of study conduction)) and maternal smoking/alcohol consumption during pregnancy. Core data of the survivor parent (including date of birth, date of cancer diagnosis, cancer diagnosis and treatment) were additionally collected from medical records by participating centers. Parental age at diagnosis was grouped as 0–4, 5–9, and 10 years or older, and the type of cancer was classified as leukaemia/lymphoma, brain tumours or extra-cranial solid tumours. We conducted telephone interviews with patients and siblings who had used ART to obtain fertility cycle data, including the type of infertility factor, type of ART, number of cycles conducted, use of fresh, cryopreserved or donor sperm/oocytes, pregnancy complications and maternal age at birth of offspring.

### 2.3. Statistical Methods

Statistical analyses were carried out with IBM SPSS Statistics software, version 27 (IBM SPSS Statistics, Chicago, IL, USA); in addition, matched-pair analysis was conducted using R software, version 4.1.2. (R Software Inc., San Francisco, CA, USA). Questionnaires lacking information on the child’s gender, age or mode of conception were excluded from analyses. Spontaneously conceived survivor offspring (*n* = 1585) and ART-conceived offspring (*n* = 74) were compared. For the additional comparison of survivor and sibling offspring, data on offspring lacking information on matching criteria (offspring gender and age, multiple births) were excluded. In total, 1294 survivor offspring were compared to 387 sibling offspring.

Perinatal and health outcomes of survivor offspring born following ART vs. spontaneous conception were analysed using descriptive statistics. *p*-values were calculated using the two-sided Chi-squared test and Pearson’s correlation for non-parametric variables, and Spearman’s correlation for parametric variables (level of significance: <0.05). Interaction effects were examined by binary logistic regression, which estimated adjusted odds ratios (ORs) and 95% confidence intervals (Cls). Binary logistic regression assessed the intervariable dependencies of the confounder’s gender, age at the time of survey, migration background, ART, preterm birth, multiple births, congenital malformations, heart defects and parental estimation of offspring health as well as parental educational attainment, smoking/alcohol consumption during pregnancy, parental age at diagnosis and type of cancer. With these independent variables, four-fold logistic regression was carried out, respectively, for the following dependent variables: preterm birth, low birth weight, congenital malformation, and congenital heart defects. Furthermore, perinatal and health outcomes of survivor offspring were compared to those of sibling offspring using a 4:1 matched-pair analysis in a case-control design. Binary logistic regression with cluster data was performed for the matched sample.

## 3. Results

### 3.1. Participants and (i) Characteristics of ART Utilisation

Overall, data from 1659 children born to childhood cancer survivors were included in our analyses, of which 74 were born following ART (4.5%). In this unmatched dataset, out of the total of 405 sibling offspring, 15 (3.7%) were born following ART. ART-conceived survivor offspring were significantly younger at the time of the survey (*p* < 0.001), more likely to be born a twin (*p* < 0.001), and none of the survivor parents reported smoking during pregnancy (*p* = 0.013) (Table 1). Paired analysis of survivor offspring matched to sibling offspring showed that survivors consumed significantly less alcohol during pregnancy (*p* = 0.002, Table 1).

Overall, 45 survivors were successfully interviewed regarding 51/74 ART-conceived offspring (68.9%, Table 2). All of these survivors had received chemotherapy, and two had also undergone radiotherapy.

In both childhood cancer survivors and siblings, male factor infertility was the more frequent reason for ART usage. The majority of survivors and siblings underwent ICSI (66.1%, 39/59) and used fresh oocytes/sperm (80.7%, 46/57). IVF and ICSI were successful after one cycle in half of the couples; however, 9.1% (4/44 pregnancies in survivors) required ≥4 cycles, whereas none of the siblings needed ≥4 cycles (Table 2).

### 3.2. Perinatal Outcomes

ART-conceived survivor offspring were born significantly more often preterm (<37 weeks of gestation, *p* <0.001) and with a birth weight below 2500 g (low birth weight, *p* < 0.001) compared to spontaneously conceived survivor offspring (Table 3). Multivariable analyses revealed that the prevalence of low birth weight and preterm birth were not associated significantly with ART. Multiple birth (*p* < 0.001), smoking during pregnancy (*p* = 0.014), congenital malformations (*p* = 0.004), older age at survey (*p* = 0.001) and solid tumours (*p* = 0.004) were associated with preterm birth in our cohort. Low birth weight was associated with preterm birth (*p* < 0.001, Table 4).

Paired analysis of survivor offspring with sibling offspring, independent of the mode of conception, revealed no significant differences in the prevalence of preterm birth and low birth weight (Table 3).

### 3.3. Prevalence of Childhood Cancer

Although ten children born to survivors (0.6%) were diagnosed with cancer (including two retinoblastomas in children with hereditary predispositions), none of the affected children were ART-conceived.

In paired analyses, only one child from the sibling offspring group was diagnosed with leukaemia (0.3%, Table 5).

### 3.4. Prevalence of Congenital Malformations and Heart Defects

Neither congenital malformations nor heart defects were more prevalent in ART-conceived survivor offspring than spontaneously conceived offspring (Table 5). Congenital malformations were shown to be associated with the male gender (*p* = 0.028, Table 4).

There was no difference in the prevalence of malformations or congenital heart defects in the survivor vs. sibling offspring population (Table 5).

## 4. Discussion

Our study examined outcomes of ART use in childhood cancer survivors and their siblings as well as perinatal and health outcomes in their offspring. Survivors of childhood cancer are at increased risk for fertility impairment, and thus, numbers of ART utilisation in this cohort have increased in recent years. In a previous study, ART was used by twice as many survivors as in the general population [30]. Our current analyses revealed that childhood cancer survivors also requested ART more often (4.5%) than their siblings (3.7%). Male factor infertility was stated as the main reason for ART by the majority of survivors; this is in line with data from the general population [35].

ART techniques continue to improve, and rising overall success rates are beneficial to former cancer patients. Currently, one-third of pregnancies are successful after one cycle, one-half after two cycles and two-thirds after four. In our study, the survivors and their siblings were comparably successful. It is reassuring to note that pregnancies were achieved after only one cycle in half of our cases, especially as ART poses an additional psychological and physical burden to both former patients and their partners. The relevance of this is emphasized by the fact that the most commonly used method in our survivors was ICSI using fresh oocytes/sperm. This requires female hormonal stimulation, which can lead to 0.5 to 5% severe ovarian hyperstimulation syndrome [36]. However, it should be noted that in 16.9% of our cases, intrauterine insemination (IUI) was successful. Although this procedure also requires the preparation of both parental partners, it is less invasive. Furthermore, a recent study revealed no increased risks of congenital defects following IUI. Still, the underlying maternal infertility presented a potential elemental risk, in addition to the risk associated with IVF [20]. Not all of our patients succeeded after only a few cycles, and a proportion required 3 or more, which can also pose a financial burden. In Germany, statutory health insurers usually pay 50% of the costs for 3 cycles [37].

The overall success of human reproduction, whether spontaneous or after IVF, also depends strongly on the mother’s age. In fact, for ART treatment, maternal age is one of the strongest predictors of success [38]. The main reasons for age-related infertility include reduced ovarian reserve and reduced oocyte/embryo competence due to age-related disorders, especially regarding an increased incidence of aneuploidy. Recent epidemiological studies confirmed that the adverse effects of chemotherapy on fertility are less severe when women seek pregnancy at a younger age [38]. In the collective studied, the average maternal age at first birth was below 30 years for both spontaneous conceptions and after ART.

To achieve higher pregnancy rates, the transfer of two or more embryos was previously the gold standard in ART. However, recent practices favour a single embryo transfer policy to avoid multiple births. The positive consequences of declining multiple birth rates after ART are decreasing perinatal risks such as preterm birth, intrauterine growth restriction and prenatal death, as well as decreasing risks for the mother, such as pre-eclampsia, diabetes and bleeding during labour [17]. Our study also showed the known increase in ART-conceived multiple births. When known confounders, including multiple births, were taken into account, perinatal outcomes, preterm births and low birth weight were no significantly different in survivor offspring, whether ART-conceived or spontaneous.

Smoking and drinking alcohol during pregnancy carries significant risks for the foetus, such as birth defects, premature birth, and low birth weight, and therefore should be avoided. We previously examined health behavior among childhood cancer survivors compared to the general population in a subgroup of this cohort. Parents who included a cancer survivor smoked less in the presence of their children. During pregnancy, mothers in cancer survivor parent couples abstained from drinking alcohol more often and smoked less [39]. Taking smoking and alcohol consumption into account as confounders, we also noticed that survivors were even more likely to abstain from smoking/alcohol during pregnancies following ART conception vs. spontaneous conception. Similarly, survivors consumed less alcohol during pregnancy than their siblings.

We did not see an increase in the prevalence of childhood cancer, congenital malformations or heart defects in ART-conceived survivor offspring compared to those conceived naturally. Similarly, we did not detect differences in survivor offspring compared to sibling offspring. A recent study also revealed no elevated risk of cancer in ART-conceived children [40].

The study setting among childhood cancer survivors in Europe posed certain limitations. Recruitment was mainly based on previous surveys which identified survivors with biological children, potentially causing a selection bias. A selection bias can generally lead to, among others, increased participation of higher-educated and female respondents in surveys. The latter was the case in our survey, as a non-responder analysis showed [32]. This approach was chosen to reduce the study burden for survivors. The questionnaire-based setting could produce a recall bias that could reduce data accuracy. However, all survivors had been treated according to standardised trial protocols, for which treatment information was available. The number of siblings and participants using ART was low. However, we had a relatively large overall sample of offspring of survivors of childhood cancer. Some data were incomplete, particularly within the ART interview. Despite these limitations, our analyses offer new insights into health issues in offspring born to childhood cancer survivors, and the high response rate reflects the strong interest shown by survivors regarding these issues.

## 5. Conclusions

Our study shows encouraging results for survivors of childhood cancer that demonstrate that the vast majority of offspring born to survivors do not experience adverse perinatal outcomes or later health problems, independently of whether a conception was spontaneous or required ART. Our findings in the survivor cohort and in comparison to their siblings support that the use of ART by childhood cancer survivors does not put offspring at additional risk for adverse health outcomes, including childhood cancer, congenital malformations or heart defects. Against the backdrop of progressively reduced toxicity regimens, our findings appear particularly reassuring for patients treated with today’s less toxic protocols for childhood cancer.

We saw an increased number of multiple births following ART to spontaneous conception in survivors. Current methods use fewer embryos per transfer to reduce these multiple birth rates, reducing associated adverse outcomes. The increasing numbers of childhood cancer survivors who turn to ART stress the importance of establishing an up-to-date information base for counselling childhood cancer patients and survivors, including this new and reassuring information.

## Figures and Tables

**Table 1:** Characteristics of participating survivor offspring, presented by mode of conception and in comparison to a sibling control group.

Characteristics	Childhood Cancer Survivor Offspring					Paired Analysis (Matched 4:1)				
	Conceived Spontaneously		Conceived by ART			Childhood Cancer Survivor Offspring		Sibling Offspring		
	Miss *	*n* (%)	Miss *	*n* (%)	*p*	Miss *	*n* (%)	Miss *	*n* (%)	*p*
Total		1585 (100.0)		74 (100.0)			1294 (100.0)		387 (100.0)	
Gender ^a^	-		-		0.436	-		-		-
Female		762 (48.1)		39 (52.7)			601 (46.4)		182 (47.0)	
Male		823 (51.9)		35 (47.3)			693 (53.6)		205 (53.0)	
Year of birth	-		-		<0.001	-		-		-
1985 to 1999		129 (8.1)		1 (1.4)			126 (9.7)		83 (21.4)	
2000 to 2009		777 (49.0)		20 (27.0)			633 (48.9)		155 (40.1)	
≥2010		679 (42.8)		53 (71.6)			535 (41.3)		149 (38.5)	
Age at time of survey ^a^	-		-		<0.001	-		-		-
Mean age (SD)		6.4 (4.9))		4.2 ((3.6)			6.7 (5.2)		8.2 (6.6)	
Median age (IQR)		5 ((6))		3 (4)			5 (8)		7 (10)	
0 to 6		975 (61.5)		59 (79.7)			727 (56.2)		189 (48.8)	
7 to 13		456 (28.8)		14 (18.9)			416 (32.1)		102 (26.4)	
≥14		154 (9.7)		1 (1.4)			151 (11.7)		96 (24.8)	
Multiple-sibling birth ^a^	-	40 (2.5)	-	22 (29.7)	<0.001	-	9 (0.7)	-	3 (0.8)	-
Migration background	1	325 (20.5)	-	13 (17.6)	0.538	1	266 (20.6)	3	92 (24.0)	0.206
Parental educational attainment	18				0.455	14		2		0.110
No professional degree		16 (1.0)		-			12 (0.9)		2 (0.5)	
In training/unspecified degree		13 (0.8)		1 (1.4)			9 (0.7)		-	
ISCED 3 to 5 (secondary/tertiary education) ^b^		916 (58.5)		38 (51.4)			750 (58.6)		208 (54.0)	
ISCED 6 to 8 (university degree or equivalent) ^c^		622 (39.7)		35 (47.3)			509 (39.8)		175 (45.5)	
Exposed to smoking during pregnancy	15	124 (7.9)	1	-	0.013	14	95 (7.4)	6	34 (8.9)	0.226
Exposed to drinking during pregnancy	22	86 (5.5)	1	4 (5.5)	0.999	15	76 (5.9)	6	49 (12.9)	0.002
Age mother at birth of first offspring	540		31		<0.001	478		197		<.001
Mean age (SD)		26.4 (4.1)		29.5 (2.9)			26.6 (4.2)		28.1 (4.5)	
Median age (IQR)		27.0 (5.0)		30.0 (2.0)			27.0 (6.0)		28.0 (6.0)	
Year of diagnosis (survivor parent)	95		2		0.894	85		-		-
<1980		42 (2.8)		1 (1.4)			38 (3.1)		-	
1980 to 1989		835 (56.0)		41 (56.9)			727 (60.1)		-	
1990 to 1999		561 (37.7)		27 (37.5)			411 (34.0)		-	
≥2000		52 (3.5)		3 (4.2)			33 (2.7)		-	
Age at diagnosis (survivor parent)	100		2		0.067	87		-	-	-
Mean age (SD)		10.7 (4.3)		9.8 (7.9)			9.8 (8.5)		-	
Median age (IQR)		11.5 (6.3)		10.6 (7.7)			10.3 (7.8)		-	
0 to 4		323 (21.8)		12 (16.7)			272 (22.5)		-	
5 to 9		378 (25.5)		12(16.7)			309 (25.6)		-	
≥10		784 (52.8)		48 (66.7)			626 (51.9)		-	
Diagnosis (survivor parent) (ICCC-3)	60		1		0.245	49		-		-
Leukemia		585 (38.4)		34 (46.6)			493 (39.6)		-	
Lymphomas		311 (20.4)		12(16.4)			243 (19.5)		-	
Brain tumors		104 (6.8)		5 (6.8)			79 (6.3)		-	
Neuroblastoma		53 (3.5)		1 (1.4)			43 (3.5)		-	
Retinoblastoma		23 (1.5)		-			21 (1.7)		-	
Renal tumors		89 (5.8)		4 (5.5)			75 (6.0)		-	
Hepatic tumors		4 (0.3)		-			4 (0.3)		-	
Bone tumors		136 (8.9)		9 (12.3)			115 (9.2)		-	
Soft tissue tumors		134 (8.8)		2 (2.7)			108 (8.7)		-	
Germ cell tumors		48 (3.1)		5 (6.8)			38 (3.1)		-	
Other malignant epithelial neoplasm		32 (2.1)		-			20 (1.6)		-	
Other neoplasm, unspecified		6 (0.4)		1 (1.4)			6 (0.5)		-	

* Miss = missing data, ART, assisted reproductive technologies; SD, standard deviation; IQR, interquartile range; ICCC-3, International Classification of Childhood Cancer, third revision (https://seer.cancer.gov/iccc/, accessed on 9 August 2022); **^a^** Matching variables for paired analysis; ^b^ including vocational schools, polytechnic schools, programs at training institutions, master craftsman training; ^c^ including bachelor‘s, master‘s, doctoral or equivalent level.

**Table 2:** Telephone interview data on ART in CCS and a sibling control group.

ART and Outcome Characteristics	Offspring Born to Survivors Using ART				
	Survivor		Sibling		
	Miss *	*n* (%)	Miss *	*n* (%)	*p*
Total of offspring born after ART		51 (100.0)		9 (100.0)	
Gender of offspring	-		-		0.855
Female		30 (58.8.)		5 (55.6)	
Male		21 (41.2)		4 (44.4)	
Infertility diagnosed in survivor/sibling	2	10 (40.0)		14 (77.8)	0.0954
Female factor		16 (32.7)		3 (33.3)	
Male factor		29 (59.2)		5 (55.6)	
Both female and male factor		4 (8.2)		1 (11.1)	
ART	1		-		0.856
IVF		9 (18.0)		1 (11.1)	
ICSI		28 (56.0)		7 (77.8)	
ICSI & TESE		3 (6.0)		-	
ICSI (with donor sperm)		1 (2.0)		-	
IUI		7 (14.0)		1 (11.1)	
IUI (with donor sperm)		2 (4.0)		-	
Use of sperm or oocytes	3		-		0.279
Fresh		37 (77.1)		9 (100.0)	
Cryopreserved before cancer treatment		3 (6.3)		-	
Cryopreserved after cancer treatment		8 (16.7)		-	
Number of IVF/ICSI treatment cycles	7		-		0.491
1		23 (52.3)		4 (44.4)	
2 to 3		17 (38.6)		5 (55.6)	
≥4		4 (9.1)		-	
Pregnancy complications	-	8 (30.8)	-	-	-
Gestational diabetes (non-insulin-dependent)		3 (37.5)		-	
Gestational diabetes (insulin-dependent)		3 (37.5)		-	
Premature contractions		1 (12.5)		-	
Vanishing twin syndrome		1 (12.5)		-	
Year of birth (survivor parent)	7		-		-
1960 to 1969		5 (11.4)		-	
1970 to 1979		19 (43.2)		-	
≥1980		20 (45.5)		-	
Age at diagnosis (survivor parent)	2		-		-
Mean age (SD)		10.7 (4.2)		-	
Median age (IQR)		12.8 (6.2)		-	
Diagnosis (grouped)	2				-
Leukemia/lymphomas		30 (61.2)		-	
Brain tumors		8 (16.3)		-	
Solid tumors		11 (22.4)		-	
Treatment including radiotherapy	6	-		-	-
Chemotherapy only		43 (95.6)		-	
Chemo- and radiotherapy		2 (4.4)		-	
Chemotherapy, radiotherapy and BMT		-		-	-
Age at birth of offspring (in total)	-		-		0.356
Mean age (SD)		32.4 (3.5)		33.3 (3.3)	
Median age (IQR)		32 (4.0)		33 (5.0)	
Partner’s age at birth of offspring (in total)	-		-		0.505
Mean age (SD)		34.0 (4.2)		34.6 (3.3)	
Median age (IQR)		34 (5.0)		35.0 (6.0)	

* Miss = missing data, ART, assisted reproductive technologies; IVF, in vitro fertilization; ICSI, intracytoplasmic sperm injection; TESE, testicular sperm extraction; IUI, intrauterine insemination; BMT, bone marrow transplant; SD, standard deviation; IQR, interquartile range.

**Table 3:** Perinatal outcomes of survivor offspring, presented by mode of conception and in comparison to a sibling control group.

Perinatal Outcome		Childhood Cancer Survivor Offspring						Paired Analysis (Matched 4:1)				
	Conceived Spontaneously (Reference Group)		Conceived by ART				Childhood Cancer Survivor Offspring (Reference Group)		Sibling Offspring			
	Miss *	*n* (%)	Miss *	*n* (%)	*OR* *95% CI*	*p*	Miss *	*n* (%)	Miss *	*n* (%)	*OR* *95% CI*	*p*
Total		1585 (100.0)		74 (100.0)				1294 (100.0)		387 (100.0)		
Gestational age	60		1			<0.001	49		14		**OR (1.062)** **(0.977–1.155)**	0.157
Mean gestational age (SD)		39.1 (2.2)		38.2 (2.2)				39.1 (2.2)		39.3 (2.3)		
Median gestational age (IQR)		40 (2)		39 (3)				40 (2)		40 (3)		
Preterm birth (<37 weeks gestation) ^a^		154 (10.1)		16 (21.9)	**OR (2.499)** **(1.401–4.459)**	<0.001		124 (10.0)		34 (9.1)	**OR (0.773)** **(0.436–1.374)**	0.381
Extremely preterm (<28 weeks gestation)		7 (0.5)		-				5 (0.4)		1 (0.3)		
Very preterm (28 to <32 weeks gestation)		20 (1.3)		2(2.7)				10 (0.8)		3 (0.8)		
Moderate preterm (32 to <37 weeks gestation)		127 (8.3)		14 (19.2)				109 (8.8)		30 (8.0)		
Term (37 to <42 weeks gestation)		1269 (83.2)		55 (75.3)				1035 (83.1)		297 (79.6)		
Post-term (42 weeks gestation or more)		102 (6.7)		2 (2.7)				86 (6.9)		42 (32.8)		
Birth weight	15		-			0.005	10		-	3	**OR (1.000)** **(1.000–1.001)**	0.037
Mean birth weight in grams (SD)		3327 (573.4)		3124 (641.3)				3339 (569.8)		3410 (585.6)		
Median birth weight in grams (IQR)		3340 (680)		3160 (888)				3350 (698)		3500 (735)		
Low birth weight (<2500 g) ^a^		103 (6.6)		13 (17.6)	**OR 3.035 (1.615–5.706)**	<0.001		83 (6.5)		26 (6.8)	**OR 1.051 (0.668–1.654)**	0.830
Extremely low birth weight (<1000 g)		7 (0.4)		-				5 (0.4)		1 (0.3)		
Very low birth weight (1000 to <1500 g)		8 (0.5)		-				6 (0.5)		3(0.8)		
Moderately low birth weight (1500 to <2500 g)		88 (5.6)		13 (17.6)				72 (5.6)		22 (5.7)		
Normal birth weight (2500 to <4000 g)		1301 (82.9)		54(73.0)				1056 (82.2)		310 (80.7)		
High birth weight (4000 g or more)		166 (10.6)		7 (9.5)				145 (11.3)		48 (12.5)		

* Miss = missing data; OR, odds ratio; CI, confidence interval; SD, standard deviation; IQR, interquartile range; ^a^ World Health Organization definitions were employed (https://www.who.int, accessed on 9 August 2022).

**Table 4:** Multivariable analysis ^a^ on the influence of different variables on the health of the child.

Characteristics (Confounders)	Preterm Birth		Low Birth Weight		Congenital Malformations		Congenital Heart Defects	
	OR (95% CI)	*p*	OR (95% CI)	*p*	OR (95% CI)	*p*	OR (95% CI)	*p*
ART	1.769 (0.864 to 3.623)	0.119	1.985 (0.806 to 4.887)	0.136	0.581 (0.077 to 4.385)	0.599	0.505 (0.060 to 4.217)	0.528
Female gender	1.105 (0.773 to 1.579)	0.584	1.131 (0.696 to 1.837)	0.620	0.529 (0.299 to 0.934)	0.028	1.756 (0.862 to 3.578)	0.121
Age at time of survey								
0 to 6	*Reference*	-	*Reference*	-	*Reference*	-	*Reference*	-
7 to 13	1.357 (0.893 to 2.061)	0.152	1.636 (0.936 to 2.858)	0.084	1.663 (0.923 to 2.996)	0.090	1.229 (0.553 to 2.731)	0.612
≥14	2.509 (1.442 to 4.366)	0.001	1.287 (0.579 to 2.864)	0.536	1.249 (0.490 to 3.182)	0.641	0.790 (0.222 to 2.811)	0.716
Multiple birth	6.111 (3.083 to 12.113)	<0.001	1.611 (0.623 to 4.170)	0.326	^b^	^b^	2.120 (0.441 to 10.181)	0.348
Exposed to smoking during pregnancy	2.083 (1.162 to 3.731)	0.014	0.697 (0.280 to 1.734)	0.437	0.668 (0.226 to 1.971)	0.465	0.523 (0.115 to 2.380)	0.401
Exposed to drinking during pregnancy	0.588 (0.238 to 1.454)	0.250	1.738 (0.64 to 4.655)	0.272	0.717 (0.165 to 3.120)	0.658	1.838 (0.518 to 6.515)	0.346
Migration background	1.208 (0.792 to 1.841)	0.380	1.311 (0.746 to 2.302)	0.347	0.891 (0.450 to 1.765)	0.741	0.737 (0.299 to 1.817)	0.508
Parental educational attainment								
No professional degree	*Reference*	-	*Reference*	-				
In training/unspecified degree	0.531 (0.037 to 7.713)	0.643	0.977 (0.29 to 33.041)	0.990	^b^		^b^	
ISCED 3 to 5 (secondary/tertiary education) ^c^	0.844 (0.157 to 4.533)	0.843	0.695 (0.059 to 8.209)	0.773	^b^		^b^	
ISCED 6 to 8 (university degree or equivalent) ^d^	0.806 (0.146 to 4.452)	0.805	0.602 (0.049 to 7.420)	0.692	^b^		^b^	
Subjective health								
Medium/poor/very poor	*Reference*	-	*Reference*	-	*Reference*	-	*Reference*	*-*
Good	0.750 (0.348 to 1.619)	0.464	0.583 (0.209 to 1.631)	0.304	0.564 (0.219 to 1.455)	0.236	0.304 (0.103 to 0.898)	0.031
Very good	0.674 (0.317 to 1.436)	0.307	0.622 (0.228 to 1.698)	0.354	0.242 (0.092 to 0.635)	0.004	0.137 (0.046 to 0.413)	<0.001
Preterm birth (<37 weeks)			26.233 (15.878 to 43.342)	<0.001	^b^		^b^	
Congenital malformations	2.778 (1.387 to 5.565)	0.004	1.326 (0.460 to 3.821)	0.602	^b^		^b^	
Congenital heart defects	2.387 (0.977 to 5.831)	0.056	2.960 (0.973 to 9.006)	0.056	^b^		^b^	
Age at time of diagnosis (survivor parent)								
0 to 4	*Reference*	-	*Reference*	*-*	*Reference*	*-*	*Reference*	*-*
5 to 9	1.119 (0.652 to 1.918)	0.683	1.086 (0.524 to 2.252)	0.825	1.043 (0.491 to 2.212)	0.913	0.724 (0.259 to 2.022)	0.538
≥10	1.040 (0.647 to 1.672)	0.872	1.019 (0.528 to 1.966)	0.956	0.688 (0.339 to 1.395)	0.300	0.799 (0.332 to 1.923)	0.616
Diagnosis (survivor parent, grouped)								
Leukemia/lymphomas	*Reference*	-	*Reference*	*-*	*Reference*	*-*	*Reference*	*-*
Brain tumors	1.182 (0.553 to 2.527)	0.667	2.305 (0.974 to 5.456)	0.058	0.544 (0.127 to 2.341)	0.414	1.468 (0.413 to 5.218)	0.553
Extracranial solid tumors	1.735 (1.190 to 2.528)	0.004	1.195 (0.709 to 2.014)	0.504	0.887 (0.488 to 1.615)	0.696	0.970 (0.449 to 2.098)	0.938

OR, odds ratio; CI, confidence interval; **^a^** health outcome of childhood cancers could not be taken into account for multivariate analyses as numbers were too small to report meaningful ORs. **^b^** Numbers (*n*) were too small to report meaningful ORs. ^c^ including vocational schools, polytechnic schools, programs at training institutions, master craftsman training. ^d^ including bachelor‘s, master‘s, doctoral or equivalent level.

**Table 5:** Prevalence of childhood cancer, congenital malformations and heart defects in CCS offspring presented by mode of conception and compared to offspring born to a sibling control group.

Health Outcome	Childhood Cancer Survivor Offspring						Paired Analysis (Matched 4:1)					
	Conceived Spontaneously		Conceived by ART				Childhood Cancer Survivor Offspring		Sibling Offspring			
	Miss *	*n* (%)	Miss *	*n* (%)	*OR* *95% CI*	*p*	Miss *	*n* (%)	Miss *	*n* (%)	*OR* *95% CI*	*p*
Total		1585 (100.0)		74 (100.0)				1294 (100.0)		387 (100.0)		
Childhood cancer (ICCC-3 *2008*)	14	10 (0.6)		-		0.491	11	8 (0.6)	2	1 (0.3)		0.408
Non-hereditary childhood cancer	15	8 (0.5)	-	-		0.538	11	6 (0.5)	2	1 (0.3)		0.585
Diagnosis of childhood cancer												
Leukemia		1 ^c^						1 ^c^		1 ^c^		
Lymphomas		1 ^c^						1 ^c^				
Brain tumors		1 ^c^						1 ^c^				
Neuroblastoma		1 ^c^						1 ^c^				
Retinoblastoma		2 ^c^						2 ^c^				
Renal tumors		2 ^c^						2 ^c^				
Hepatic tumors		-						-				
Bone tumors		-						-				
Soft tissue tumors		1 ^c^						-				
Germ cell tumors		-						-				
Other malignant epithelial neoplasm		-						-				
Other neoplasm, unspecified		-						-				
Congenital malformations (Q00-Q99, ICD-10 *2016*)	18	90 (5.7)	-	2 (2.7)		0.267	15	60 (4.7)	4	15 (3.9)		0.720
Diagnosis	7		-				16		-			
Nervous system		1 (1.1)		1 (33.3)				2 (3.3)		-		
Eye, ear, face and neck		7 (7.8)		-				6 (10.0)		-		
Circulatory system ^a^		31 (34.4)		1 (33.3)				12 (20.0)		1 (33.3)		
Respiratory system		-		-				-		-		
Cleft lip and cleft palate		4 (4.4)		-				3 (5.0)		1 (33.3)		
Digestive system		6 (6.7)		1 (33.3)				7 (11.7)		-		
Genital organs		6 (6.7)		-				4 (6.7)		-		
Urinary system		7 (7.8)		-				5 (8.3)		1 (33.3)		
Musculoskeletal system		23 (25.6)		-				17 (28.3)		-		
Other congenital malformations		4 (4.4)		-				3 (5.0)		-		
Chromosomal abnormalities, not elsewhere classified		1 (1.1)		-				1 (1.7)		-		
Number of congenital malformations reported ^b^		90 (5.7)		3 (4.1)				60 (4.7)		3 (0.8)		
Number of children with congenital malformations		83 (5.3)		2 (2.7)				55 (4.3)		3 (0.8)		
Congenital heart defects (Q20-28, ICD-10 *2016*)	28	38 (2.4)	1	1 (1.4)		0.558	43	17 (1.4)	11	6 (1.6)		0.711
Diagnosis	-		-				-		-			
Cardiac chambers and connections		-		-				-		-		
Cardiac septa		14 (31.8)		1 (100.0)				4 (21.1)		2 (25.0)		
Pulmonary, tricuspid valves, aortic & mitral valves		5 (11.4)		-				-		1 (12.5)		
Other congenital malformations of the heart		16 (36.4)		-				13 (68.4)		5 (62.5)		
Great arteries and great veins		9 (20.5)		-				2 (10.5)		-		
Other malformations of the peripheral vascular system		-		-				-		-		
Other malformations of the circulatory system		-		-				-		-		
Number of reported congenital heart defects ^b^		44 (2.8)		1 (1.4)				19 (1.5)		8 (2.1)		
Numbers of children with congenital heart defects		38 (2.4)		1 (1.4)				17 (1.4)		6 (1.6)		

* Miss = missing data; CI, confidence interval; ICCC-3, International Classification of Childhood Cancer, third revision (https://seer.cancer.gov/iccc/), accessed on 9 August 2022; SD, standard deviation; IQR, interquartile range; ICD-10, International Statistical Classification of Diseases, 10th revision; OR, odds ratio. ^a^ Diagnosis of congenital malformations of the circulatory system are reported in detail in congenital heart defects. ^b^ Children with multiple diagnoses appear more than once in the table. ^c^ Numbers (n) were too small to report meaningful percentages.

## Data Availability

The data presented in this study are available on request from the corresponding author.
